# Attenuation of Immunogenicity in MOG-Induced Oligodendrocytes by the Probiotic Bacterium *Lactococcus* Sp. PO3

**DOI:** 10.3390/medicina59101731

**Published:** 2023-09-27

**Authors:** Ashraf Khalifa, Hairul-Islam Mohamed Ibrahim, Abdullah Sheikh, Hany Ezzat Khalil

**Affiliations:** 1 Biological Science Department, College of Science, King Faisal University, P.O. Box 400, Al-Ahsa 31982, Saudi Arabia; 2 Botany and Microbiology Department, Faculty of Science, Beni-Suef University, Beni-Suef 62511, Egypt; 3 Molecular Biology Division, Pondicherry Centre for Biological Sciences and Educational Trust, Pondicherry 605004, India; 4 Camel Research Center, King Faisal University, P.O. Box 400, Al-Ahsa 31982, Saudi Arabia; 5 Department of Pharmaceutical Sciences, College of Clinical Pharmacy, King Faisal University, Al-Ahsa 31982, Saudi Arabia; 6 Department of Pharmacognosy, Faculty of Pharmacy, Minia University, Minia 61519, Egypt

**Keywords:** cow’s milk, *Lactococcus*, probiotics, neuroinflammatory diseases, oligodendrocyte

## Abstract

*Background and Objectives*: Milk is healthy and includes several vital nutrients and microbiomes. Probiotics in milk and their derivatives modulate the immune system, fight inflammation, and protect against numerous diseases. The present study aimed to isolate novel bacterial species with probiotic potential for neuroinflammation. *Materials and Methods*: Six milk samples were collected from lactating dairy cows. Bacterial isolates were obtained using standard methods and were evaluated based on probiotic characteristics such as the catalase test, hemolysis, acid/bile tolerance, cell adhesion, and hydrophobicity, as well as in vitro screening. *Results*: Nine morphologically diverse bacterial isolates were found in six different types of cow’s milk. Among the isolates, PO3 displayed probiotic characteristics. PO3 was a Gram-positive rod cell that grew in an acidic (pH-2) salty medium containing bile salt and salinity (8% NaCl). PO3 also exhibited substantial hydrophobicity and cell adhesion. The sequencing comparison of the 16S rRNA genes revealed that PO3 was *Lactococcus raffinolactis* with a similarity score of 99.3%. Furthermore, PO3 was assessed for its neuroanti-inflammatory activity on human oligodendrocyte (HOG) cell lines using four different neuroimmune markers: signal transducer and activator of transcription (STAT-3), myelin basic protein (MBP), glial fibrillary acidic protein (GFAP), and GLAC in HOG cell lines induced by MOG. Unlike the rest of the evaluated neuroimmune markers, STAT-3 levels were elevated in the MOG-treated HOG cell lines compared to the untreated ones. The expression level of STAT-3 was attenuated in both PO3-MOG-treated and only PO3-treated cell lines. On the contrary, in PO3-treated cell lines, MBP, GFAP, and GLAC were significantly expressed at higher levels when compared with the MOG-treated cell lines. *Conclusions*: The findings reported in this article are to be used as a foundation for further in vivo research in order to pave the way for the possible use of probiotics in the treatment of neuroinflammatory diseases, including multiple sclerosis.

## 1. Introduction

Milk is a rich source of nutrients and diverse microbiomes. Milk and its products are habits for beneficial bacteria known as probiotics, which have a key role in immunomodulatory, anti-inflammatory, and antimicrobial action against several diseases. They have been part of human food and culture for a long time due to their use in medicine, food manufacturing, and preservation [1]. Their effectiveness depends on their quantity in food (10^6^–10^7^), with 10^9^ per gram of colony forming units (CFUs) recommended per day [2,3]. Certain factors such as animal health, hygienic milking practice, transportation, and storage conditions influence the microflora of the milk [4]. The composition of the microbiota also varies according to the milk and its products. Bacterial genera including *Bifidobacterium*, *Enterococcus*, *Lactobacillus*, *Leuconostoc*, *Pediococcus*, *Propionibacterium*, and *Streptococcus* and yeast genera such as *saccharomyces* and *Aspergillus* are found in dairy products [4,5,6]. The microbiota from cow’s milk has been reported, via culture-independent methods, to contain 146 different bacterial strains belonging to the predominant taxa of *Bifidobacterium*, *Corynebacterium*, *Enterococcus*, *Pseudomonas*, *Propionibacterium*, *Streptococcus*, and *Staphylococcus* [7,8,9,10,11]. The colostrum has been reported to have major taxa including *Bacteroidales*, *Clostridiales*, *Prevotella*, *Pseudomonas*, *Ruminococcaceae*, and *Staphylococcus* [12]. Hence, it is important to characterize the potential probiotic bacteria and their role in immunomodulatory effects on neuroinflammation.

Lactic acid bacteria are widely used in dairy industries to ferment food products through acidification or low pH to preserve them. Moreover, they contribute proteolytic, lipolytic, and organoleptic characteristics to the fermented foodstuffs, such as flavor and texture [13,14]. Generally, low temperatures below 4 °C are preferred for raw milk storage; however, this negatively affects the mesophilic bacteria and favors pathogenic bacteria such as psychotrophic bacteria [13,15,16]. To this end, the pre-maturation method was followed by increasing the storage temperature to favor the desired microbiota, such as *Lactococci*, which is helpful in developing various cheese products [15,17]. One of the studies reported that *Lactococcus* (38.6%) and *Streptococcus* quantity increased significantly after 24 h of refrigeration [18].

Gut microbiota plays a key role in regulating the interactions among the central nervous system, endocrine glands, and inflammatory markers [19,20]. They regulate the interaction between the intestinal barrier and the brain when administered adequately [21]. Furthermore, the gut microbiome may influence the neurotransmitters and cytokine production to boost the host immune system [22]. They synthesize short-chain fatty acids (SCFAs) which aid in host immunity and lower inflammation [23,24]. The neurological disorder known as multiple sclerosis was also treated with probiotics [25]. Several ailments such as diabetes [26], inflammatory bowel disease [27,28], and neuronal inflammatory disorder [29,30] were improved by the immunomodulatory mechanisms of specific probiotic organisms.

*Lactococcus raffinolactis* and its other members belong to the family *Streptococcaceae*, and they are used in different industries for meat preservation, starters, and flavoring purposes [31]. There are many studies on the isolation and antimicrobial features of probiotic bacteria such as *Lactobacillus* and *Lactococcus*. However, few studies correlate the probiotic effect on neurological inflammation. Therefore, this study aims to focus on the probiotic potential of *Lactococcus raffinolactis* and its role in the modulation of neuroinflammation. It has recently been reported that *Lactococcus lactis* displayed a significant positive impact in an LPS-induced depression-like model in mice [32], alleviated depressive and anxiety symptoms in mice [33], attenuated acute psychological stress in healthy Japanese men [34], and prevented the migration of cells into the spinal cord in experimental autoimmune encephalomyelitis [35]. Despite the above-mentioned beneficial effects of probiotic bacteria, little is known about the group of bacteria in cow milk in the Al-Ahsa region. Therefore, the current work is designed to obtain new probiotic bacteria species with potential therapeutic neuroinflammatory benefits. Six separate cow’s milk samples were used to identify nine morphologically diverse bacterial strains. PO3 was extracted from 25th-week breastfeeding milk and was shown to have probiotic characteristics. Additionally, the role of the potential probiotic bacteria in modulating the neuroinflammation disorder was examined.

## 2. Materials and Methods

### 2.1. Sample Collection and Handling Procedures

For the bacterial analysis, 6 samples of lactating dairy cows (one-week lactating milk (Colostrum) and 25th-week lactating milk) were collected from January to March 2023 in the mid-winter season (Dairy Processing Department, Agricultural and Veterinary Research Station, King Faisal University, Hofuf, Eastern province, Saudi Arabia). A total of 400–500 mL of milk was collected in sterilized containers. The samples were labeled and stored at 4 °C until use.

### 2.2. Isolation of Beneficial Bacteria from Cow Milk

Isolation of beneficial bacteria from lactating cow milk was performed using the serial dilution method. A total of 1 mL of cow milk was added to 24 mL of de Man, Rogosa, and Sharpe (MRS) broth, and diluted milk (0.2 mL) was spread and plated on an MRS agar plate for 48 h. The single colonies were selected and stored in 30% glycerol stock with 10% skim milk with consecutive −70 °C storage conditions [36].

### 2.3. Catalase and Hemolytic Activity

The Gram-positive isolates from cow milk were screened for catalase activity using the H_2_O_2_ slide drop assay. The hemolytic activity of active isolates was examined on Colombia blood agar (Himedia, Mumbai, India) [37].

### 2.4. Identification Methods

#### 2.4.1. Phenotypic Characterization

Colony morphology, cell shape, Gram staining, catalase activity, and hemolytic activity were investigated after 48 h on MRS agar plates at 30 °C. The isolates were checked for physiological traits, including growth in 8% (*w*/*v*) NaCl and at pH 3.0 according to the method described earlier by [38]. Based on phenotypic parameters and biochemical characteristics, active selected isolates were further screened for hydrophobic characters, bile salt digestion, and cholesterol assimilation.

#### 2.4.2. Bile Salt Hydrolase (BSH) Activity

The activity of BSH was determined following the method mentioned earlier by [39], with slight modifications. Briefly, 20 µL of actively growing culture was carefully spotted on MRS agar amended with calcium chloride (0.037% (*w*/*v*)) and taurodeoxycholic acid (0.5% (*w*/*v*)). Plates were incubated anaerobically at 37 °C for 72 h. Zone forming colonies or white colonies indicated signs of BSH activity. The baseline was MRS agar that had not been changed.

#### 2.4.3. Cell Surface Hydrophobicity

The ability of bacterial cells to adhere to the mucus environment was achieved by using octane solvent in an in vitro environment (Octan: Merck Co., Roway, NJ, USA). The assay was performed according to a previously described method [40].

The hydrophobicity index (HBI) was calculated as follows:

HBI = [(A1 − A2)/A1] × 100.

#### 2.4.4. Cholesterol Assimilation

The ability of beneficial bacteria to assimilate cholesterol was assessed in MRS broth supplemented with cholesterol-polyethylene glycol (PEG) 600 (Sigma, Ronkonkoma, NY, USA) at a final concentration of 100 µg mL^−1^. The MRS-cholesterol-PEG 600 mixture was incubated at 37 °C for 24 h with each 1% (*v*/*v*) inoculum. The turbidity of MRS broth was measured by CFU methods. The assimilation of cholesterol was detected by centrifuging the broth at 4000 rpm for 10 min, and the supernatant was collected for absorbance analysis using a microplate spectrum reader (570 nm). The concentrations of cholesterol were compared to the absorbance of the standard curve, with cholesterol concentrations ranging from 0 to 500 µg/mL in MRS (R^2^ = 0.987). The efficiencies of beneficial bacteria to cholesterol assimilation in MRS were determined as follows:

Cholesterol assimilated (µg mL^−1^)
= (Cholesterol [µg mL^−1^]) 0 h − (Cholesterol [µg mL^−1^]) 24 h

% Cholesterol assimilated
= (Cholesterol assimilated [µg mL^−1^]/Cholesterol [µg mL^−1^] 0 h) × 100%

### 2.5. In Vitro Screening of Probiotic Properties

Preparation of bacterial cell suspensions was performed as described by Pithva et al. [41] to evaluate the attributes of the probiotics. The chosen bacteria were cultured in MRS broth at 30 °C for 12 h. Overnight cultures were centrifuged at 9000× *g* for 10 min at 4 °C and resuspended in sterile physiology saline (0.88%) to adjust the cell culture (OD600 = 1 and 10^9^ CFU/mL).

#### Acid and Bile Tolerance

The simulated gastric acid and intestinal bile fluids were prepared as described previously [40]. The acid and bile broth suspensions were incubated at 37 °C for 3 h with stirring at 200 rpm. Cell viability was assayed by CFU/mL.

The percentage of bacterial survival was calculated as follows:

Cell survival = CFUassay/CFUcontrol × 100,

where CFU assay represents CFUs after incubation and CFU control represents the CFUs after incubation in PBS. Experiments were performed as three independent experiments.

### 2.6. Effect of Probiotics on Neuroanti-Inflammatory Activity

#### Cell Culture and Cell Differentiation

Human oligodendrocyte (HOG) cell lines (PCBS laboratory, India) were routinely grown at 37 °C in Dulbecco’s Modified Eagle’s Medium (DMEM) with serum supplemented with 10% (*v*/*v*) fetal bovine serum (FBS) and 100× antimycotic solution under humidified and 5% CO_2_ conditions.

### 2.7. Adhesion Assay

Human oligodendrocyte cells were used to study the adherence characteristics of PO3 (the selected strain with probiotic potentialities). HOG cells (1.5 × 10^5^ cells) were cultivated for 72 h in cell culture inserts (Boyden chamber). After 72 h, the solution containing 5 mM sodium butyrate was replaced, and cells were cultured for 96 h to promote differentiation. HOG cells were aspirated with sterile PBS and bacterial cell suspension and were resuspended in antibiotics-free DMEM. After 90 min in a 37 °C 5% CO_2_ incubator, each cell suspension was plated on wells. Unbound bacterial cells were removed by sterile PBS aspiration and were subsequently lysed with 0.05% Triton X-100 solution. Spot plate counts on MRS agar were performed after 48 h of incubation at 37 °C to determine the total number of attached bacteria. The following equation was used to determine the adhering abilities of the chosen isolate:

Percentage of adhering cells (%) = Nt/N0 × 100

where Nt = the number of LAB cells adhering to Caco-2 cells.

N0 = the total number of inoculated LAB cells

### 2.8. Measurement of NO Production

HOG cell lines induced with MOG at 1 µg/mL (4 × 10^4^ cells/well) were inoculated in 48-well plates for 24 h. The serum-free DMEM (1% FBS) was stored overnight before treatment. The starved cells were pre-incubated with 1 µg/mL MOG for 4 h, and the active isolate PO3 was inoculated and co-cultured for 24 h. The supernatant was examined for NO production, which was determined using an NO detection kit (Griess reagent kit) in accordance with the manufacturer’s protocol.

### 2.9. Cytokine Assays

The HOG cells were activated with 1 µg/mL of MOG for 4 h. Subsequent treatment with probiotic isolates was followed by measurement of the production of pro-inflammatory cytokines, including IL-1, IL-6, and IFN-γ. The cell-free supernatant was prepared using RIPA lysis buffer, and the lysates were used to quantify the cytokines using ELISA kits (Invitrogen, Thermo Fisher Scientific, Vienna, Austria; Cayman, CA, USA) in accordance with the manufacturing guidelines. The amounts of cytokines on the plates were measured using an automated ELISA plate reader at 450 nm (BioTek Instruments, Winooski, VT, USA). The standard was used for each cytokine marker with R^2^ = 0.985.

### 2.10. RT-PCR Analysis of Genes on the Inflammatory Signaling Pathway

Total RNA was extracted from MOG-induced HOG cell lines in accordance with the instructions provided by the manufacturer (Roche, Germany). The cDNA synthesis kit was used to generate complementary DNA (cDNA), and the process was carried out in compliance with the guidelines provided by the manufacturer. The qPCR primers that are shown in Table 1 were selected with the use of the Primer Bank website, which was accessed at http://pga.mgh.harvard.edu/primerbank (accessed on 15 March 2023).

In order to determine the optimal annealing temperature for each primer set, gradient PCR was used. The quantity of mRNA produced by the genes under investigation was measured using the detection instrument ABI step one plus (Applied Biosystems, Foster City, CA, USA) and was supplied with SYBR Green master mix manufactured by Amplicon Bio in Denmark. In order to ensure accuracy, all reactions were carried out twice. The relative amount of gene expression was calculated using the formula RQ = 2^−ΔΔCt^ [16], which was utilized for the comparative CT approach. In order to standardize the results, GAPDH, a housekeeping gene, was chosen as the appropriate internal control gene.

### 2.11. Genotypic Characterization

The isolates of 16S rRNA gene sequences were amplified using PCR following the method mentioned earlier by the authors of [43]. PCR products were sent to the “Macrogen” for sequencing using universal primers 27 F (5′-GAGTTTGATCCTGGCTAG-3′) and 1525 R (5′-AGAAAGGAGGTGATCCAGCC-3′). The EzBiocloud program was used to estimate the values of similarity in sequences between PO3 and its other similar recognized species [44]. MEGA 7 used the neighbor-joining (NJ) method to generate a phylogenetic tree [45,46]. The confidence values for each branch in the phylogenetic tree were determined using a bootstrap approach with 1000 repetitions [38]. The detected sequences were deposited in the NCBI GenBank.

### 2.12. Statistical Analysis

All the tests were carried out three times to ensure accurate data, and the findings are presented as the mean along with the standard deviation (SD). The results of tests on acid and bile tolerance and adhesion capacity were analyzed using ANOVA and the SPSS 22.0 program. When comparing mean values at a significance level of *p* < 0.05, Duncan’s Multiple Range Test (DMRT) was used as the appropriate statistical tool. Welch’s *t*-test, with a significance threshold of *p* < 0.05, was used to evaluate the immunomodulatory effects of the treatment.

## 3. Results

Nine morphologically distinct bacterial isolates were obtained from six cow’s milk samples. There were four (PN1, PN2, PN3, and PN4) and five isolates (PO1, PO2, PO3, PO4, and PO5) obtained from the 1st and 25th week of lactating cows, respectively (Table 2). As can be seen in Table 1, the isolates were diverse in terms of cell shape (cocci and rod) and growth at 8% NaCl and pH 3. All but PN4 and PO1 grew at 8% NaCl. Only PN1, PN2, PO1, PO2, and PO3 displayed growth at a pH of 3. All isolates were catalase-negative; however, PN4 was catalase-positive, reacting with oxygen bubbles when H_2_O_2_ was added. PN3 and PN4 exhibited hemolytic activities, whereas the remaining isolates did not.

**Figure 1:**
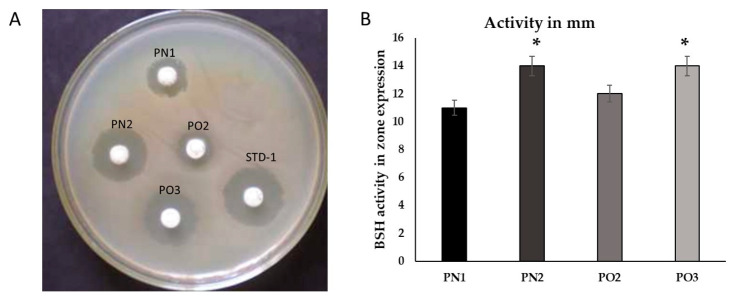
Bile salt hydrolase activity: The zone of hydrolase indicates enzyme reactivity against bile salt in MRS media. MRS media was prepared and supplemented with 0.8% bile salt for plate preparation. (**A**): The disc impregnated with different cow milk isolates (PN1, PN2, PO2, and PO3). The standard probiotics (STD-1) was *Lactobacillus casei*. (**B**): The BSH activity of the four bacterial isolates in terms of zone expression. The zone indicates the hydrolysis of bile sale and is expressed in mm. The data are shown as mean ± SD. Variation in lettering indicates statistical significance (*p* < 0.05). * *p* < 0.05 represents significance between the groups.

Four isolates, PN1, PN2, PO2, and PO3, were selected for further screening using bile salt hydrolase (BSH) activity, and the results are presented in Table 3 and Figure 1. The BSH activity of PN1 and PO2 was 11 mm and 13.6 mm, respectively. Comparable results (11.6 mm and 13.8) for BSH activity were obtained for PN2 and PO3 (Figure 1).

### 3.1. Bacterial Adhesion

**Figure 2:**
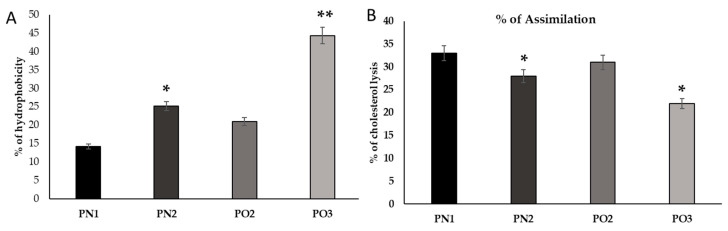
Hydrophobicity data on cholesterol assimilation ability are represented as the mean ± SD. The MRS media were supplemented with filter-sterilized cholesterol-polyethylene glycol (PEG) 600 (Sigma) at a final concentration of 100 µg mL^−1^ and incubated anaerobically at 37 °C for 24 h. The degradation of cholesterol was quantified using left over MRS media using the formula. (**A**): the hydrophobicity percentages of the cow milk isolates (PN1, PN2, PO2, and PO3). (**B**): the assimilation of cholesterol by the bacterial isolates. The data are shown as mean ± SD. Variation in lettering indicates statistical significance (*p* < 0.05). * *p* < 0.05 represents significance between the groups. ** *p* < 0.01 represents significance between the groups.

**Figure 3:**
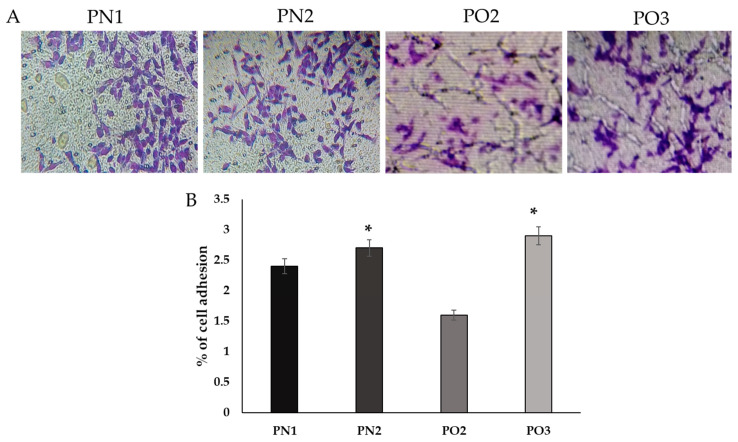
Percentage of selected isolates adhering to HOG cell lines. (**A**): phase contrast inverted microscopic images of selected isolates that were enumerated by bacterial cultures and interpreted as the percentage of adherence relative to the control. (**B**): the percentage of cell adhesion for the cow milk isolates (PN1, PN2, PO2, and PO3). The data are shown as mean ± SD. * *p* < 0.05 represents significance between the groups.

The capacity of probiotics to attach to epithelia is assessed in vitro by measuring the cells’ hydrophobicity at the surface for certain solvents (octane). This determines whether or not the probiotics are able to attach to the epithelia. It was discovered that the isolate has a significant degree of hydrophobicity (Figure 2). The results indicated that PO3 had the highest adhesion percentage (3%) towards octane. PO2 displayed the lowest adhering percentage (1.5) (Figure 3).

**Figure 4:**
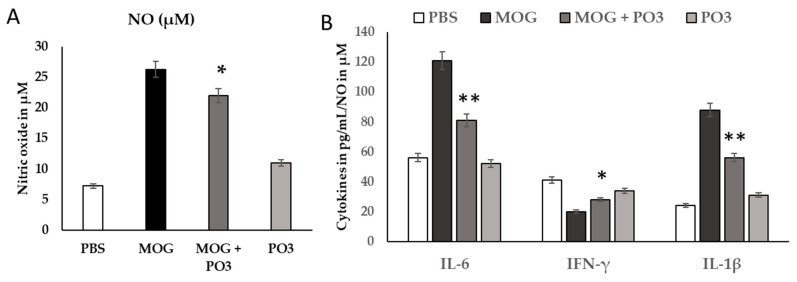
Effect of PO3 on inflammatory markers of (**A**) nitric oxide and (**B**) IL-6, IFN-γ, and IL-1β cytokines in HOG cell lines induced by MOG. These markers were quantified in MOG-induced HOG cell lines and PO3 treated for 12 h. The treated cells were homogenized after 12 h of induction using RIPA lysis buffer. Invitrogen and Cayman ELISA kits were used for quantification of the cytokines and chemokines. Estimation of the quantity of NO was performed using a kit from Invitrogen. The optical variation was observed at 450 nm using a microplate reader, and values were expressed in µM. Cytokines IL-6 and IFN-γ were quantified using the Cayman kit. All data were collected from three individual experiments, pooled, and expressed as mean ± SD (*p* < 0.05). * *p* < 0.05 represents significance compared to the MOG vs. MOG + PO3 group. ** *p* < 0.01 represents significance between the groups.

The findings for the pro-inflammatory cytokines (IL-6 and IFN-γ) are shown in Figure 4. Noticeable variations were observed in IL-6 and IFN-γ activity between the treated and control groups in MOG-treated cell lines (Figure 4). In comparison to the MOG group, a significant reduction in IL-6 levels was observed in the PO3-treated groups (*p* < 0.01). Unlike IL-6, a comparative evaluation of IFN-γ levels in MOG-treated cells was compared to the negative control ones. The presence of PO3 significantly reduced the increased levels of NO seen in MOG-treated cells (Figure 4A).

**Figure 5:**
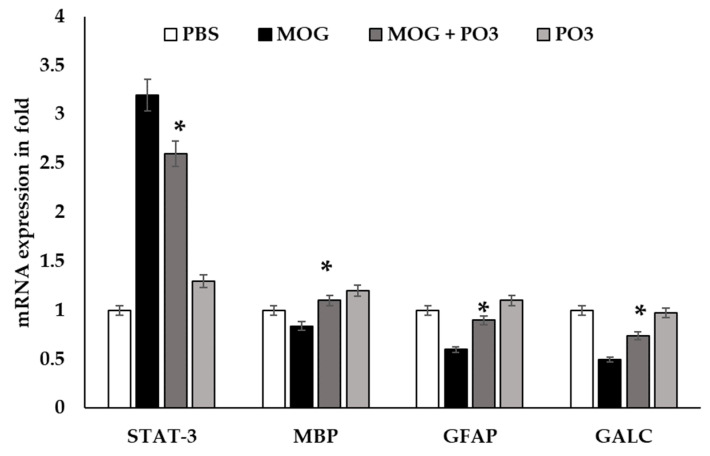
Effect of PO3 on neurological immune markers (STAT-3, MBP, GFAP, and GALC in HOG cell lines induced by MOG (1 µg)). The expression levels of the markers were estimated in HOG cell lines treated with MOG and those treated with PO3 for 18 h. The treated cells were homogenized after 12 h of induction using RIPA lysis buffer. The cytokines and chemokines were quantified using Invitrogen and Cayman ELISA kits. mRNA expression was expressed as fold units. GAPDH was used as the internal control to nullify the expression of targets. All data were collected from three individual experiments, pooled, and expressed as mean ± SD (*p* < 0.05). * *p* < 0.05 represents significance compared to the MOG vs. MOG + PO3 group.

The impact of PO3 on the induction of neuroimmune markers (STAT-3, MBP, GFAP, and GALC) in HOG cell lines by MOG (1 µg) was studied and the results are presented in Figure 5. Unlike the rest of the evaluated neuroimmune markers, STAT-3 levels were elevated in the MOG-treated HOG cell lines compared to the untreated ones. The expression level of STAT-3 was attenuated in both PO3-MOG-treated cell lines and those only treated with PO3. In contrast, in PO3-treated cell lines, MBP, GFAP, and GALC were significantly expressed at higher levels when compared with the MOG-treated cell lines.

### 3.2. Molecular Identification of PO3 Using Sequencing of the 16S rRNA Gene

**Figure 6:**
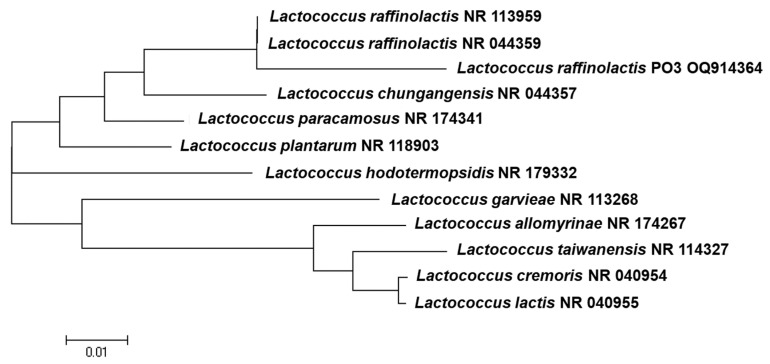
A phylogenetic tree based on the 16S rRNA gene sequences of PO (OQ914364)3 and the cluster showing a higher similarity sequence with *Lactococcus* genus groups from NCBI GenBank. The horizontal scale bar denoting the similarity between isolates expresses a 0.01 difference in nucleotide position.

In this study, the new isolate PO3 was identified through comparative analysis of the isolates with the bioinformatic details from the GenBank sequence (Figure 6). The isolate PO3 was identified as *Lactococcus raffinolactis* at a similarity level 99.3%. The submitted 16S rDNA sequences received the accession number for PO3 (OQ914364). The neighbor-joining method revealed the active isolate was *Lactococcus raffinolactis*, with the 0.001 bootstrap closer to *Lactococcus raffinolactis* NR044359 (Figure 6).

## 4. Discussion

Disturbance of the gut–brain axis, a bidirectional relationship between the microbiota in the digestive tract and the brain, has been linked to neuroinflammatory response and neurological disorders [29,30]. Cow milk offers a suitable nutritional medium for many microorganisms, including probiotics, due to its high content of essential molecules including carbohydrates, proteins, lipids, vitamins, and minerals. The aim of the current work was to isolate novel probiotic bacterial strains with potential therapeutic impacts on neuroinflammation. Six different samples of cow’s milk were used to obtain nine bacterial isolates, each of which was unique morphologically. Among these isolates, PO3 was isolated from 25th-week lactating milk and exhibited probiotic attributes. PO3 was Gram-positive, and rod cells grew in a highly acidic medium (pH 3), bile salt, and in a saline environment (8% NaCl). PO3 also showed a high percentage of hydrophobicity and cell adhesion. The isolate modulated the levels of the proinflammatory markers in the cell lines. In accordance with the sequence comparison of the 16S rRNA gene, PO3 was proven to be *Lactococcus raffinolactis* with a similarity level of 99.3%. A variety of characteristics, both in vitro and in vivo, must be met before a microbe can be considered a probiotic; they include resistance to the hydrophobicity of the cell surface for adhesion, the presence of bile salts and acid, and negative catalase reactions [47]. It has been reported that the aggLr gene on the chromosome encodes the new aggregation component of *L. raffinolactis* BGTRK10-1, AggLr. Domain analysis (AggLr comprises collagen-binding and CnaB domains with an LPxTG motif on the C terminus) indicated that AggLr is a member of the SFCBAF protein family [48].

PO3 was unable to generate the catalase that is required to produce oxygen and water by splitting hydrogen peroxide. This finding also implies that PO3 can live in environments with extremely low concentrations of oxygen. These findings were comparable to those that were published by Saroha et al. (2023) [49], who described a Gram-positive, catalase-negative strain of *Limosilactobacillus walteri* as a novel probiotic bacterial strain [49]. Screening bacteria for the products of hemolysis, which are responsible for the death of host cells, is very necessary in order to assure the safety of the isolate. PO3 did not exhibit any hemolytic activity, which suggests that it is believed to be a safe strain that does not pose any damage to the host and is suitable for use in the production of probiotics.

Hydrophobicity at the surface of cells is measured in vitro to evaluate the ability of probiotics to attach to epithelia. The ability of the probiotics to bind to the epithelia depends on this. According to the findings, PO3 exhibited the maximum adhesion towards xylene (3%). Selecting probiotic bacteria for use as therapeutic agents requires consideration of their ability to attach to the gut. Colonization of the human gut by probiotics is believed to be needed for them to perform their therapeutic effects, such as the avoidance of enteropathogenic bacteria [50]. The coaggregation of probiotic strains results in a barrier that hinders colonization by infectious microbes. Adherence to epithelial cells seems to require autoaggregation of probiotic strains. Milerien et al. (2023) also observed that *Lactococcus lactis* has very high hydrophobicity, as measured by microbial adherence to xylene, with a value of 89.3% [51,52].

A crucial biological barrier that a probiotic strain has to overcome to reach its target is the acid that is found in the stomach, as well as the bile salts that are found in the intestine. In environments with a low pH, metabolic activity may be restricted, and the growth and survival of probiotics may be hindered. Therefore, the ability of probiotic strains to tolerate acid, gastric juice, and bile is critical to their capacity for survival and proliferation in the gastrointestinal system. In the research that we conducted, we found that PO3 was resistant to bile salt, acidic pH, and stimulated gastric juice. These findings are comparable to those obtained with the probiotic *Lactobacillus* sp. strain in the earlier investigation [53,54].

The modulatory impact of PO3 on the expression of myelin basic protein (MBP) neuroimmune markers (signal transducer and activator of transcription (STAT-3), glial fibrillary acidic protein (GFAP), and GLAC) was studied in HOG cell lines induced by MOG. Unlike the rest of the evaluated neuroimmune markers, STAT-3 levels were elevated in the MOG-treated HOG cell lines compared to the untreated ones. The expression level of STAT-3 was attenuated in both PO3-MOG treated cell lines and those only treated with PO3. In contrast, in PO3-treated cell lines, MBP, GFAP, and GLAC were significantly expressed at higher levels when compared with the MOG-treated cell lines. It has been reported that Hsp65-producing *Lactococcus lactis* inhibits experimental autoimmune encephalomyelitis by preventing cell migration into the spinal cord [35]. Furthermore, other probiotic species such as *Bacillus amyloliquifaciens*-supplemented camel milk suppress the neuroinflammation of autoimmune encephalomyelitis by regulating inflammatory markers [29], as well as by regulating SOX5/miR-218 axis orchestration in mouse models [30]. STAT-3 diminished apoptosis and polarized the OLs in LPS-induced immunogenicity, which resulted in defective regulation of MBP and GFAP in OLs. The lineage of OL cells was progressed by GFAP and MBP and was significantly expressed in lymphoid cells.

The activation of STAT-3 signaling may be triggered by a variety of stimuli and is crucial for the functions of many distinct types of cells [55]. By manipulating the STAT-3 gene in astrocytes, we demonstrated the protective role of reactive astrocytes in limiting myelin injury. It has been reported that probiotic strains may indirectly minimize *H. pylori* infection by stimulating phagocytoses, natural killer (NK) cells, DCs, and antibody and anti-inflammatory cytokines [56]. STAT-3 regulates neurogenerative markers (MBP in myelin sheaths), GFAP, and GALC in neuroglial cells. One can speculate that, in our study, *Lactococcus* sp. PO3 abrogated STAT-3 and activated the neuroprotective markers to improve cell communication and the functionality of the blood–brain barrier. In support of that, *L. rhamnosus* and *L. acidophilus* have been found to block STAT-3 and NF-B activation in dendritic cells via upregulation of certain anti-inflammatory markers (IL-10 and Foxp3+ Treg) [57].

## 5. Conclusions

The six cow’s milk samples yielded nine morphologically distinct bacterial isolates. PO3, from 25th-week lactating cow milk, was a probiotic. PO3 was Gram-positive, and rod cells were developed in highly acidic medium (pH 3), with bile salt, and in a saline environment (8% NaCl). PO3 was hydrophobic and cell-adhesive. The isolate altered cell line proinflammatory markers. PO3 was shown to be *Lactococcus raffinolactis* through 99.3% 16S rRNA gene sequence similarity. PO3 displayed substantial modulatory impacts on the immune markers in the MOG-induced cell lines. The results presented herein are considered a base for further in vivo investigation in order to open the door for potential application of the probiotic to combat neuroinflammation disorders.

## Figures and Tables

**Table 1:** A list of the primers used to target the immune regulatory markers.

Primer Name	Forward	Reverse	PCR Product Size	Ref.
STAT-3	CTTTGAGACCGAGGTGTATCACC	GGTCAGCATGTTGTACCACAGG	189	[42]
MBP	ATTCACCGAGGAGAGGCTGGAA	TGTGTGCTTGGAGTCTGTCACC	245	[30]
GFAP	CTGGAGAGGAAGATTGAGTCGC	ACGTCAAGCTCCACATGGACCT	144	https://www.origene.com/catalog (accessed on 15 March 2023)
GalC	ATCTCTGGGAGCCGATTTCCTC	CCACACTGTGTAGGTTCCAGGA	135	https://www.origene.com/catalog (accessed on 15 March 2023)
GAPDH	GTCTCCTCTGACTTCAACAGCG	ACCACCCTGTTGCTGTAGCCAA	188	[30]

**Table 2:** Isolation and physiological characterization of isolates from milk samples.

	One-Week Lactating Milk				25th-Week Lactating Milk				
No of Isolates	PN1	PN2	PN3	PN4	PO1	PO2	PO3	PO4	PO5
Shape of the cell	Rod	Rod	Cocci	Cocci in chain	Cocci	Rod	Rod	Rod	Cocci
Growth (at 8% NaCl)	+	+	+	−	−	+	+	+	+
Growth (at pH 3)	+	+	−	−	+	+	+	−	−
Catalase	−	−	−	+	−	−	−	−	−
Hemolysis activity	−	−	+	+	−	−	−	−	−

Data on growth ability are expressed as follows: +, positive reaction; −, negative reaction. Catalase and hemolysis activity and similarly expressed.

**Table 3:** Gastric acid and bile juice tolerance of selected probiotics.

Selected Probiotic Strains	pH 3	pH 4	Bile 0.3%	Bile 0.6%
PN1	5.6 ± 0.7	6.12 ± 0.2	9.2 ± 0.57 *	8.8 ± 0.3 *
PN2	7.9 ± 0.2 *	6.5 ± 0.3	9.1 ± 0.9	8.7 ± 0.2
PO2	5.5 ± 0.1	6.4 ± 0.4	8.8 ± 1.1	7.8 ± 0.3
PO3	6.64 ± 0.3 *	6.9 ± 0.2 *	9.25 ± 0.7 *	8.9 ± 0.6 *

Data are expressed as the mean ± SD. * *p* < 0.05, significantly different from the negative control.

## Data Availability

Available from the corresponding author upon request.
